# Evaluation of illness severity of neonate infectious pneumonia and neurobehavioral development through ultrasonography under adaption algorithm

**DOI:** 10.12669/pjms.37.6-WIT.4883

**Published:** 2021

**Authors:** Kangkang Meng, Chao Ying, Jianwei Ji, Lianfang Yang

**Affiliations:** 1 Kangkang Meng, Attending Physician. Department of Neonatology, Yiwu Central Hospital, Yiwu, 322000, China; 2 Chao Ying, Attending Physician. Department of Neonatology, Yiwu Central Hospital, Yiwu, 322000, China; 3 Jianwei Ji, Attending Physician. Department of Neonatology, Yiwu Central Hospital, Yiwu, 322000, China; 4 Lianfang Yang, Attending Physician. Department of Neonatology, Yiwu Central Hospital, Yiwu, 322000, China

**Keywords:** Ultrasound imaging, SS technology, IPN, Newborn, AD

## Abstract

**Objectives:**

To explore the diagnostic effect of ultrasound imaging on the illness severity, and to analyze neurobehavioral development of neonates with Infectious Pneumonia (IPN), Self- Adaptation (SD), and Spatial Smoothing (SS) technologies were adopted to build SDSS. Then, the WFFSF algorithm based on Wiener Filtering (WF) and Feature Space Fusion (FSF) and the SNRP-FSF algorithm based on Signal-to-noise ratio post-filtering (SNRP) and FSF were introduced for comparison.

**Methods:**

One hundred and thirty-two neonates were divided into group without respiratory failure (S1) and respiratory failure group (S2). The study was conducted from March 2018 to July 2020. According to scoring systems for neonatal critically illness, they were divided into non-severe group (W1), severe group (W2), and extremely-severe group (W3). According to the Scale of Child Development Center of China (CDCC), they were divided into a normal neurobehavioral developmental group (P1) and an abnormal neurobehavioral developmental group (P2).

**Results:**

The normalized mean square distance l and normalized mean absolute distance f of SDSS algorithm were significantly lower than that of WFFSF algorithm and SNRP-FSF algorithm, and the peak signal-to-noise ratio (PSNR) was significantly higher than that of WFFSF algorithm and SNRP-FSF algorithm (P<0.05). The lung ultrasound score (40.62±7.22%) of S1 was greatly higher than S2 group (28.47±6.29%) (P<0.05); the lung ultrasound score (39.13±8.25) in W1 was greatly higher than W2 (27.28±6.39) and W3 groups (14.33±7.03); neonates in group W2 had higher lung ultrasound scores than W3 (P<0.05), and lung ultrasound scores in P1 (42.57±8.58) was greatly higher than that the P2 group (26.49±6.09).

**Conclusion:**

In contrast with traditional algorithms, the SDSS algorithm based on AD has a better reconstruction effect on neonatal IPN ultrasound images. The lung ultrasound score can clearly indicate the severity of the disease and neurobehavioral development of neonate IPN, and the lung ultrasound score is negatively correlated with the severity of the child’s disease and the abnormality of neurobehavioral development.

## INTRODUCTION

At present, imaging techniques are widely used in the diagnosis of lung diseases, such as X-ray, CT, and MRI.^1^-^3^ However, exposing the newborn to ionizing radiation will affect their growth and development to a certain extent.^4^-^6^ Therefore, it is necessary to find a method that can be used for the early diagnosis of pneumonia. Hence, the ultrasound score can be used in the semi-quantitative assessment of the severity of lung diseases.^7^,^8^ In order to improve the diagnostic effect of ultrasound imaging, intelligent algorithms are used to segment the ultrasound images.^9^ Among them, the use of adaptive algorithm to reconstruct the ultrasound image can effectively reduce the artifacts and noise in the ultrasound image.^10^

In summary, an ultrasound image reconstruction algorithm SDSS was constructed based on SD and SS technology. Then, it was applied to ultrasound imaging diagnosis of neonates with IPN. By comparing the lung ultrasound scores of neonates in S1 and S2, W1, W2, and W3, and P1 and P2, the diagnostic effect of ultrasound imaging on the severity of the disease was comprehensively evaluated.

## METHODS

**Fig.1:**
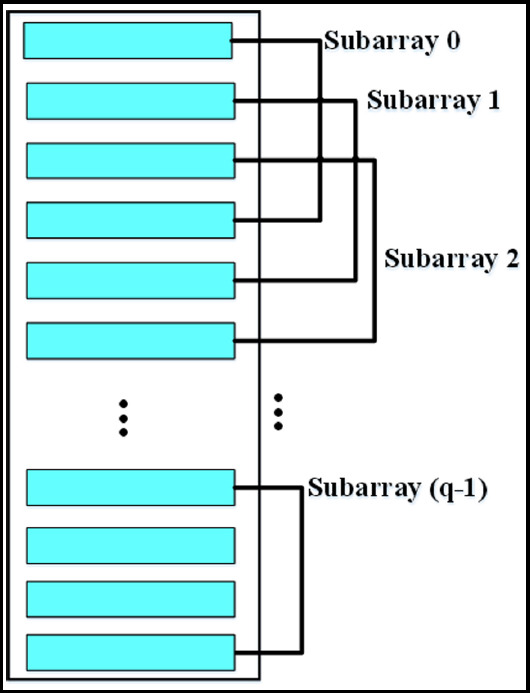
Sub-array division of ultrasonic linear array sensor.


One hundred thirty two full-term newborns who were diagnosed with IPN in our hospital from March 2018 to July 2020 were selected as the research subjects. They were divided into S1 and S2 groupsThree senior physicians adopted the two-lung twelve-zone scoring method to diagnose lung lesions.In An ultrasonic linear array sensor with M independent array elements was set, and the sensor was divided into q sub-arrays. Fig-1.

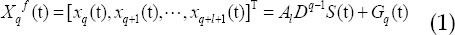


In which, *l* indicated the length of the sub-array, *A_l_* indicated the flow matrix of the reference array, *A_l_*=[*a_l_*(*θ*_1_),*a_l_*(*θ*_2_),*a_l_*(*θ*_3_),...,*a_l_*(*θ*_k_)], *S(t)* indicated the echo signal matrix, 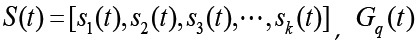 indicated the noise matrix, 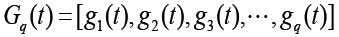 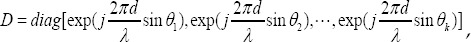, D represented the noise variance matrix. Then the sampling covariance matrix of the ultrasonic linear array sensor can be expressed as follows.


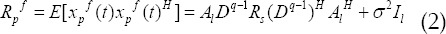



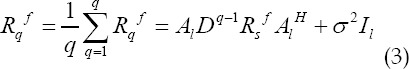


The backward SS covariance matrix of the sensor was as follows.


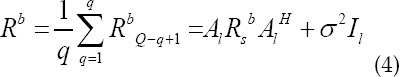


In which, 
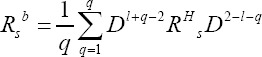
. After calculating the arithmetic average according to equations (3) and (4), the forward and backward SS covariance matrix of the ultrasonic sensor can be obtained as follows.


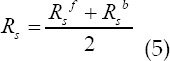


In which, *R_s_* represented the forward and backward SS covariance matrix of the ultrasonic sensor, *R_s_^f^* represented the forward SS covariance matrix of the ultrasonic sensor, and *R_s_^b^* represented the backward SS covariance matrix of the ultrasonic sensor. The above was the ultrasound image reconstruction algorithm based on SD and SS technology, which was set as SDSS.

Evaluation index of reconstruction quality. The WFFSF algorithm based on Wiener Filtering and Feature space fusion and the SNRP-FSF algorithm based on signal-to-noise ratio post-filtering and Feature space fusion were introduced in the study. They were compared to the SDSS algorithm proposed in the study. The normalized mean square distance *l*, normalized mean absolute distance f, and PSNR were selected to evaluate the reconstructed image quality of different algorithms.

I. *l*: it is used to indicate the deviation between the reconstructed image and the original image. The higher the value, the greater the deviation between the two.


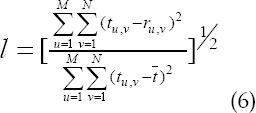


In which, *t_u,v_* represented the pixel density of row U and column V of the original image. *r_u,v_* represented the pixel density of row U and column V of reconstructed image. 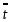 represented the average density of the original image. *M* × *N* represented the number of pixels in the image.

II. *f*: it is used to represent the absolute error between the reconstructed image and the original image. The larger the value is, the larger the error is.


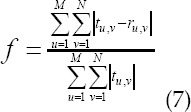


**III. PSNR:** the image quality is evaluated mainly by calculating the pure mathematical statistics of the error between pixels. The larger the value is, the less the image distortion is.


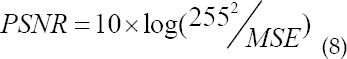



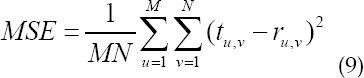


In which, *MSE* denoted mean square error.

(5) Statistical methods. The data were processed by SPSS19.0, mean ± standard deviation (x± s) was adopted to express the measurement data, and the count data was expressed as percentage (%). The lung ultrasound scores of neonates in groups S1 and S2, neonates in groups W1, W2, and W3, and neonates in groups P1 and P2 were compared by analysis of variance. With P<0.05, the difference was statistically significant.

## RESULTS

**Fig.2:**
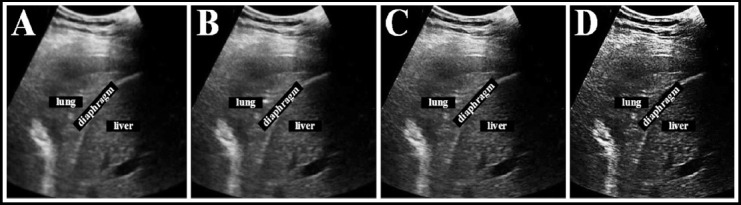
Comparison of the reconstruction effects of three algorithms on a male neonate’s lung ultrasound image (age 10 days, weight 3.17kg). ***Note:*** A was the original image; B was the WFFSF algorithm; C was the SNRP-FSF algorithm; D was the SDSS algorithm.

**Fig.3:**
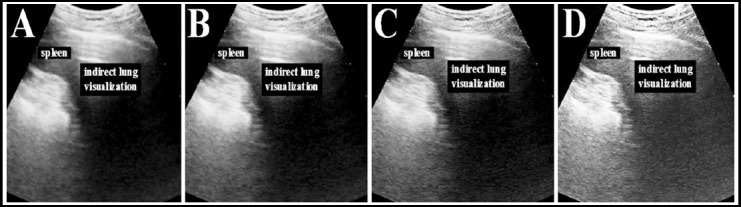
Comparison of the reconstruction effect of three algorithms on a female neonate’s lung ultrasound image (age 8 days, weight 2.85kg). (A, B, and C had the same indications with Fig.3).

Comparison of mean square error convergence of the algorithm. In Fig.2 and Fig.3, it was evident that the lung ultrasound reconstructed image by SDSS algorithm was more clearly visible, the contrast was more obvious, the artifacts and noise were also minimal, and the overall effect was better. Descriptive statistics of basic data. As shown in Table-I:

**Table-I:** Descriptive statistics of basic data of neonates.

*Variable*	*Classification*	*Sample size (person)*	*Proportion (%)*
Gender	Male	74	56.06
	Female	58	43.94
Age (days)	<9	70	53.22
	≥9	62	46.78
Weight (kg)	<3	67	51.08
	≥3	65	48.92
Ways to produce	Caesarean section	82	61.92
	Normal delivery	50	38.08
Mother’s childbearing age (years)	<30	75	56.72
	≥30	57	46.28
Gestational age (weeks)	<34	28	21.43
	34-37	65	49.36
	>37	39	29.21
Abnormal umbilical cord	Yes	37	28.15
	No	95	71.85

**Fig.4:**
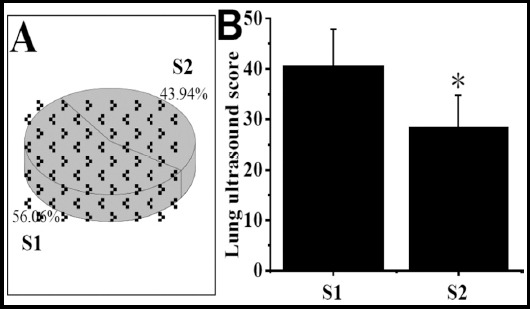
Comparison of lung ultrasound scores in S1 and S2. ***Note:*** A was the proportion of S1 and S2 groups; B was comparison of lung ultrasound score. * indicated that the difference was obvious in contrast with the S1 group (P<0.05).

Comparison of lung ultrasound scores in S1 and S2. As shown in Fig.4 below, the proportion of neonates in S1 (56.06%) was higher than S2 (43.94%). The lung ultrasound score of the neonates in the S1 was 40.62±7.22%, and that in the S2 was 28.47±6.29%. The lung ultrasound scores of neonates in S1 were greatly higher than S2 group (P<0.05).

**Fig.5:**
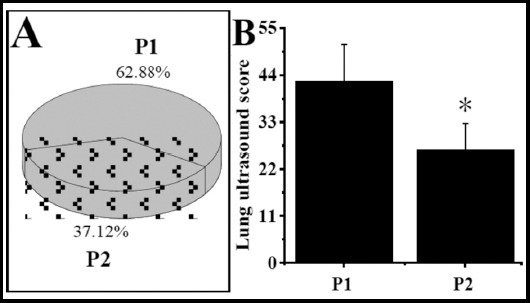
Comparison of lung ultrasound scores between P1 and P2. ***Note:*** A was the proportion of P1 and P2 groups; B had the same indication with Fig.4. * indicated that the difference was obvious in contrast with the P1 group (P<0.05).

Comparison of lung ultrasound scores between P1 and P2As shown in Fig.5 below, the proportion of neonates in the P1 (62.88%) was greatly higher than P2 group (37.12%), and the lung ultrasound score (42.57±8.58) of P1 was greatly higher than that of the neonates in the P2 group (26.49)±6.09) (P<0.05).

## DISCUSSION

Neonatal pneumonia mainly refers to the pulmonary inflammation caused by pathogen infection in the womb, during delivery, or after birth, and it is the main cause of neonatal death.^11^,^12^ Because the lung of newborn is not completely developed, the mucous membranes of the trachea and bronchi are soft and rich in blood vessels, plus the cleaning ability is poor, so they are prone to infection and respiratory failure.^13^ Clinically, X-ray examination is used to diagnose neonatal pneumonia based on clinical symptoms and physical signs.^14^,^15^ However, the clinical symptoms and signs of neonatal pneumonia are not obvious, and X-ray examination may cause cancer, gene mutation, and chromosomal mutation. As a result, the application of X-ray in the diagnosis of neonatal pneumonia is limited. Ultrasound examination is characterized by non-radiation, simple operation, and affordable price. When used in the examination of lung diseases, it can dynamically monitor the outcome of lung inflammation.^16^ Artifacts or noise in ultrasound images can be attributed to individual differences, improper operation, or environmental factors.^17^ Therefore, to determine the disease process using ultrasound images, image preprocessing is required. In this study, first, an SDSS image reconstruction algorithm based on SS technology and AD technology was proposed, and used to reconstruct neonatal ultrasound images, together with other adaptive algorithms. Then, the effects of different algorithms were compared. The results showed that, compared with WFFSF and SNRP-FSF algorithms,^18^,^19^ the SDSS algorithm had higher definition and contrast after reconstruction, and effectively removed artifacts and noise in the image. The main pathological feature of neonatal pneumonia is the exudation of inflammatory mediators, and the liquid and gas in the alveoli can cause artifacts.^20^ The SDSS algorithm can remove artifacts and noise in ultrasound images, indicating that the SDSS algorithm can be applied to reconstruct ultrasound images.

## CONCLUSION

In the study, the SS technology and AD were combined to construct an ultrasound image reconstruction algorithm SDSS, which was applied to the ultrasound imaging diagnosis of newborns with IPN. The lung ultrasound score can clearly indicate the severity of the disease and neurobehavioral development of newborns with IPN. Besides, the lung ultrasound score is negatively correlated with the severity of the child’s disease and the abnormality of neurobehavioral development.

### Authors’ Contribution:

**KM:** Conceived the study, literature review, analysis of data and drafting of the paper.

**JJ & LY:** Helped in design, data collection, article drafting & critical revision.

**CY:** Takes the responsibility and is accountable for all aspects of the work in ensuring that questions related to the accuracy or integrity of any part of the work are appropriately investigated and resolved.

## References

[1] Vishnu Bhat B, Adhisivam B (2018). Can We Reduce the Duration of Antibiotic Therapy for Neonatal Pneumonia?. Indian J Pediatr.

[2] Zar HJ, Andronikou S, Nicol MP (2017). Advances in the diagnosis of pneumonia in children. BMJ.

[3] Biagi C, Pierantoni L, Baldazzi M, Greco L, Dormi A (2018). Lung ultrasound for the diagnosis of pneumonia in children with acute bronchiolitis. BMC Pulm Med.

[4] Xiao TT, Jin M, Ju R, Yang S, Gao SQ (2018). Value of bedside lung ultrasound in the diagnosis of neonatal pneumonia. Zhongguo Dang Dai Er Ke Za Zhi.

[5] Sharma D, Farahbakhsh N (2019). Role of chest ultrasound in neonatal lung disease:A review of current evidences. J Matern Fetal Neonatal Med.

[6] Eslamy HK, Newman B (2011). Pneumonia in normal and immunocompromised children:An overview and update. Radiol Clin North Am.

[7] Soldati G, Demi M, Smargiassi A, Inchingolo R, Demi L (2019). The role of ultrasound lung artifacts in the diagnosis of respiratory diseases. Expert Rev Respir Med.

[8] Picano E, Scali MC, Ciampi Q, Lichtenstein D (2018). Lung Ultrasound for the Cardiologist. JACC-Cardiovasc Imag.

[9] Lyu J, Ling SH, Banerjee S, Zheng JJY, Lai KL (2019). 3D Ultrasound Spine Image Selection Using Convolution Learning-to-Rank Algorithm. Annu Int Conf IEEE Eng Med Biol Soc.

[10] Al Mukaddim R, Meshram NH, Varghese T (2020). Locally optimized correlation-guided Bayesian adaptive regularization for ultrasound strain imaging. Phys Med Biol.

[11] Niedzwiecka T, Patton D, Walsh S, Moore Z, O'Connor T (2019). What are the effects of care bundles on the incidence of ventilator-associated pneumonia in paediatric and neonatal intensive care units?A systematic review. J Spec Pediatr Nurs.

[12] Zar HJ, Andronikou S, Nicol MP (2017). Advances in the diagnosis of pneumonia in children. BMJ.

[13] Xiao TT, Jin M, Ju R, Yang S, Gao SQ, Jiang Y (2018). Value of bedside lung ultrasound in the diagnosis of neonatal pneumonia. Zhongguo Dang Dai Er Ke Za Zhi.

[14] Hiles M, Culpan AM, Watts C, Munyombwe T, Wolstenhulme S (2017). Neonatal respiratory distress syndrome:Chest X-ray or lung ultrasound?A systematic review. Ultrasound.

[15] Le Roux DM, Zar HJ (2017). Community-acquired pneumonia in children - a changing spectrum of disease. Pediatr Radiol.

[16] Rea G, Sperandeo M, Di Serafino M, Vallone G, Toma P (2019). Neonatal and pediatric thoracic ultrasonography. J Ultrasound.

[17] Seyman EE, Bornstein N, Auriel E, Cohen O, Nissel T (2019). Assessment of carotid artery ultrasonography in the presence of an acoustic shadow artifact. BMC Neurol.

[18] Chen Y, Tu Z, Kang D, Chen R, Bao L (2021). Joint Hand-Object 3D Reconstruction from a Single Image with Cross-Branch Feature Fusion. IEEE T Image Process.

[19] Doris MK, Otaki Y, Krishnan SK, Kwiecinski J, Rubeaux M (2020). Optimization of reconstruction and quantification of motion-corrected coronary PET-CT. J Nucl Cardiol.

[20] Van Sloun RJG, Demi L (2020). Localizing B-Lines in Lung Ultrasonography by Weakly Supervised Deep Learning, In-Vivo Results. IEEE J Biomed Health.

